# Primary school children exhibit socioeconomic inequalities in their usual beverage consumption: baseline assessment of the DRINK trial

**DOI:** 10.1186/s13690-025-01730-0

**Published:** 2025-10-09

**Authors:** Lucille Desbouys, Wassila Assakali, Isabelle Thiébaut, Katia Castetbon

**Affiliations:** 1 Research Center in “Epidemiology, Biostatistics and Clinical Research”, School of Public Health, Université libre de Bruxelles, CP598, Route de Lennik 808, Brussels, 1070 Belgium; 2 Club Européen Des Diététiciens de L’Enfance (CEDE), Ath, 7800 Belgium

**Keywords:** Beverage consumption, Sweetened beverages, Water consumption, Usual consumption, Socioeconomic disparities, Children, School interventions

## Abstract

**Background:**

Promoting favourable beverages to children remains a public health priority, and schools are essential in reducing nutritional disparities. The study aimed to describe the baseline characteristics of schools and children included in the DRINK trial, and to examine disparities in children’s usual beverage consumption.

**Methods:**

The study is a cross-sectional analysis of data collected at baseline of the DRINK cluster randomised controlled trial. Children aged 8–11 years from 46 French-speaking primary schools in Belgium were invited to complete 4-day diaries and questionnaires during the spring 2021. Usual consumption of total beverages, water, sweetened beverages (SB), and milk was estimated by correcting the data for within-person variation using the Statistical Program to Assess Dietary Exposure (SPADE) and compared between subgroups using bootstrapping to generate 95% CIs.

**Results:**

A diverse range of schools and children participated in the trial. Of the 3,631 students, 2,427 completed a valid diary and child questionnaire, including fewer from Brussels than Wallonia. Children usually consumed 1,109 ml/day (95% CI: 1,092–1,132) of total beverages, including 677 ml/day (660–699) of water, and 345 ml/day (331–359) of SB. Socio-economic disparities were observed in all types of beverage consumption, mostly related to the parental education, and most strikingly for the water intake.

**Conclusion:**

Despite the challenges of participation, partly due to the Covid-19 pandemic, inclusion in the DRINK trial provided a wide diversity of school populations, which will ultimately be considered when interpreting the trial findings. The overall high consumption of SB and the socio-economic disparities, particularly in water consumption, emphasize the need to reinforce nutrition interventions among disadvantaged children.

**Trial registration:**

The DRINK trial was registered on 25 May 2021 in the ISRCTN Registry (ISRCTN99843102).

**Table:** 

Text box 1. Contributions to the literature
- The DRINK trial's methodology, which includes an age-adapted four-day diary and robust statistical modelling, demonstrates the feasibility of estimating the usual beverage consumption in children aged 8-11.
- Baseline analysis highlights the high usual intake of sweetened beverages in Belgium and confirms, in line with national dietary surveys, pronounced socioeconomic disparities, particularly in water consumption.
- Moreover, capturing the diversity of participating schools and students will enhance the further interpretation of the trial results.
- Such findings emphasise the influence of the socioeconomic environment on healthier beverage behaviours, thereby reinforcing the relevance of evaluating school-based nutrition interventions.

## Background

The high consumption of sweetened beverages (SB) among children and adolescents remains a public health concern [1, 2], due to their significant contribution of added sugar in the diet [3], which may impair their future health [4]. Belgian adolescents are particularly at risk: in 2018, students in French-speaking schools of Belgium were among the five highest European consumers of SB, with 29.4% drinking one or more sugary soft drinks daily [2].

Socio-economic and cultural disparities have been reported in SB consumption among children and adolescents. SB intake is likely to be higher when socio-economic status, parental education, and household wealth are lower [5]. Among Belgian adolescents, being an immigrant and living in a less affluent family have also been associated with a higher prevalence of daily SB consumption [6]. Furthermore, over the last two decades, although total intake and daily consumption of SB among children and adolescents have decreased overall, disparities have tended to widen in some high-income countries, such as the U.S. [7] and Belgium [2]. This rise in SB social inequalities emphasizes the need for efficient health promotion actions among youth.

Schools are key settings for health promotion among children from diverse backgrounds, though school-based interventions have shown only moderate effectiveness in reducing SB consumption [8–10]. To enhance impact, high-quality evaluations are required [10]. Additionally, integrating sustainability and environmental concerns into messaging may positively influence SB consumption [11, 12].

In this framework, the DRINK trial (“Decreasing consumption of sugar-sweetened beverages and Raising tap water consumption through Interventions based on Nutrition and sustainability for Kids”) was set up in spring 2021 to evaluate the long-term effectiveness of nutrition- and sustainability-based interventions on the reduction in SB intake and on the increase in tap water consumption in 3rd to 6th grade children (8 to 11 years of age) [13]. One of its features is its implementation in all types of school settings, with the premise that most children would benefit from the intervention.

Even with a robust methodology and close monitoring, ensuring participation in a real-life trial remains a critical challenge. Beyond the distal circumstances such as the COVID-19 pandemic, school recruitment is hindered by logistical constraints, parental mistrust, and measurement-related obstacles [14]. The contextual complexities underscore the need to assess implementation, uptake, and setting to support interpretation of findings [15]. Ensuring that participation is not limited to the most available or motivated schools is essential. A detailed characterisation of participating schools and students enhances the interpretation of the trial results and provides insights into how schools and student characteristics –particularly the socioeconomic status– may independently influence the children’s eating behaviour [16].

Another key issue lies in the reliable estimations of beverage consumption collected among primary school children. Achieving good data quality requires appropriate instruments. For instance, a study conducted in 2012 and aiming to evaluate the total fluid intake of Belgian children aged 8 to 13 years through a 7-day diary, reported a median total beverage intake of 864 ml/day, concluding to an insufficient fluid intake [17]. In comparison, the 2014 Belgian food consumption survey (BNFCS) which used repeated 24-h recalls and propensity questionnaires, i.e. a method considered more robust and less prone to underreporting, estimated a higher usual intake of around 1,100 g/day for children aged 10–13 years [18]. Using reliable methods of collection and analyses is essential to estimate the usual beverage consumption, in total and for the relevant beverage categories.

This study valuably contributes to the existing literature by documenting the conditions surrounding the inclusion stage of a real-life, school-based nutritional trial. By offering contextual insights, it will support the interpretation of the broader intervention outcomes.

Based on the DRINK trial baseline data, the objectives assessed were twofold: 1) to describe the schools and children’s inclusion and baseline features, and 2) to study disparities in the children’s usual beverage consumption. Given the conditions of this real-life trial and the instruments used – validated questionnaires and 4-day beverage diary– we hypothesized that a diversity of school and child profiles was obtained, and that usual beverage consumption levels were plausible, with marked socioeconomic disparities, particularly for sweetened beverages.

## Methods

### Study settings

The DRINK trial is a cluster randomised trial developed in 2021, the clusters being French-speaking primary schools in Belgium. The sampling was stratified on region (Brussels/Wallonia) and on three stratum of the school socio-economic index (SEI). Details of the trial protocol have been described elsewhere [13]. The trial was registered on 25 May 2021 in the ISRCTN Registry (ISRCTN99843102).

**Fig. 1:**
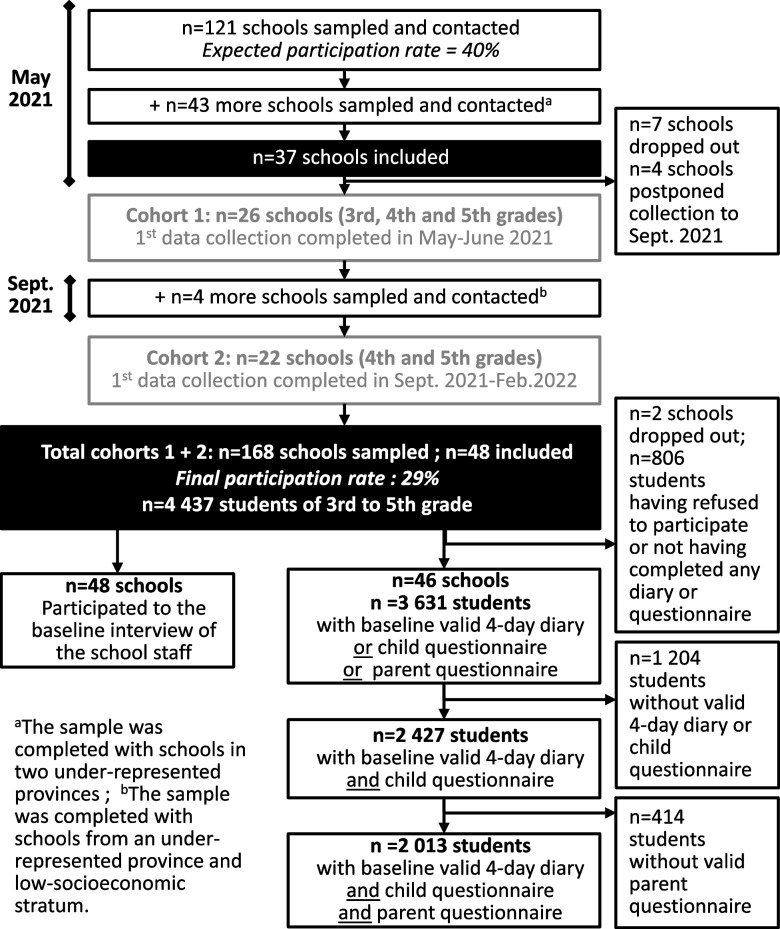
Flow chart of school sampling, inclusion, and participation in the DRINK^*^ trial, Belgium, 2021–22. ^*^Decreasing consumption of sugar-sweetened beverages and Raising tap water consumption through Interventions based on Nutrition and sustainability for Kids

The recruitment of the schools began in May 2021, before the end of the school year. From the 1,379 eligible schools, 168 schools were sampled and contacted (Fig. 1). The participation rate being below the expected 40%, the initial sample of schools had to be supplemented to achieve the number of 48 schools required for the trial, resulting in a final participation rate of 29%. In addition, because some schools asked to postpone the first data collection due to insufficient staff to manage it, the recruitment period was extended to the beginning of the following school year. Therefore, two cohorts were formed, of 26 and 22 schools respectively. As the questionnaires required a certain level of reading and understanding, the grade of the included children was adapted to the cohort: children in the first cohort were in grades 3 to 5 and those in the second cohort were in grades 4 to 5. In both cohorts, children were aged from 8 to 11. The baseline data collection period ran from May 2021 to January 2022.

The baseline data collection included students, parents, teachers, and school staff, after which schools were randomised into four intervention groups, following a factorial plan: (1) Nutrition-based interventions; (2) sustainability-based interventions; (3) both nutrition-and sustainability-based interventions; (4) control schools. Interventions were set up from January 2022. The paper here is focused on pre-intervention analysis.

### Data collection at baseline

The first trial judgement criterion being the change over time in mean SB consumption, children completed a 4-day diary to collect information on all the beverages consumed during over four randomly selected consecutive whole days, including weekends, at any place. This booklet included different stickers to help the children describe the moment of the day (10 stickers), the type (13) and the amount (22) of each beverage consumed. Children were also encouraged to write the brand, taste, low sugar content allegation, any complementary information about the beverage, and any additions such as cocoa, sugar, or flavoured syrup. One day of the 4-day diary was calibrated against a 24-h recall administered by a paediatric dietitian, considered as the reference method.

Children and parents self-completed paper questionnaires during class and at home respectively. Information about drink and food consumption behaviours, health status, environmental risk perception and behaviours, demographics, socio-economic status, living conditions, wellbeing, and social relationships, was collected. Details on the use of validated tools and references have been given in the trial protocol’s publication [13]. To adapt the wording, length and clarity, child questionnaire and 4-day diary were pre-tested among students in the same age group as the trial, schooled in volunteer schools of diverse socio-economic levels. These schools were not subsequently included in the trial.

School staff participated in a 1-h face-to-face interview conducted by a dietician. The main topics addressed were health promotion actions related to nutrition and sustainability implemented in the school, access to tap water, sanitary facilities, impact of the COVID-19 pandemic, and parental involvement in school activities (details available in the trial protocol [13]).

During the period of school recruitment, a short semi-structured questionnaire on the main reasons for non-participation was sent to each school that refused to participate. Those who had not answered this online questionnaire were recontacted by phone and by e-mail to complete it.

### Beverage consumption

Beverage 4-day diaries were manually entered into a dedicated Excel® form by trained Public Health students. At least the type and quantity of beverage had to be mentioned to encode the given consumption. Problematic diaries, such as less than three beverages consumed in a single day, no sticker for the type of beverage or indication for the quantity, or all lines of the booklet filled incorrectly, were set aside for further assessment by the research team to determine their exclusion.

Information related to beverage quantity, container, taste, brand, sugar content, and additions for each beverage consumed was checked and harmonized. When the container was not indicated by the student and the sticker for the type of beverage could be interpreted as a volume, a mean quantity of beverage was assigned. For example, the sticker for “bottled water” was represented by a plastic bottle, thus the quantity corresponding to an average small bottle was attributed, i.e. 330 ml. Then, before computing mean consumptions, quantities exceeding 2,000 ml for each beverage were treated on a case-by-case basis. The most frequent situations that could be corrected were when a child indicated a number of glasses or bottles instead of a number of mouthfuls; confused ml, cl, dl, etc.; or chose a sticker clearly inconsistent with the other occurrences in the diary. If such quantities were eventually not plausible, the whole day of consumption or the whole 4-day diary was removed. Diaries with less than two days of consumption were also discarded. At this stage, out of a total of 2,982 diaries encoded, 103 diaries (3.5%) were excluded.

Six beverage categories were defined based on the type of beverage reported by the child, combined with brand, taste and sugary addition: 1) water; 2) SB including light soda; 3) non-sweetened milk; 4) coffee, tea; 5) soup. Due to the confusing allegations about the sugar content of the beverage packaging (“low sugar”, “low calorie” but sweetened drinks coexisting with “zero” and “light” drinks effectively without added sugar), particularly for children of these ages, the information about low sugar content, that could be indicated using a sticker “light”, could not be used. Therefore, it was not possible to confidently distinguish between sugar-sweetened beverages and genuinely “light” sodas, thus an overall SB group was formed.

On this basis, a mean daily consumption for total beverages, water, SB, and non-sweetened milk was computed for each student. A student was considered as an over-reporter and removed from the analyses if the mean water consumption exceeded two standard deviations (SD) from the mean (> 2,026 ml/day), as well as if the mean total beverage consumption exceeded two SD (> 2,366 ml/day). At this step, an additional 8% of diaries were excluded, after which no implausible outliers remained.

### Description variables

Region, school size, network, and SEI were included in the official sampling list provided by the Ministry of Education. The SEI is a score based on the socio-economic characteristics of the families of the children attending the school. The tertiles of SEI were used to identify “low”, “moderate” and “high” SEI.

Children answered six questions of the family affluence scale (FAS), a validated index of family wealth [19]. A FAS score ranging from 0 to 13 was computed and divided in three quintile-based categories: “low” corresponded to the 20% of children with the lowest FAS score, “medium” to the 60% with the intermediate score, and “high” to the 20% with the highest score. The migration status was determined on the child’s and parents’ self-declared countries of birth (or one parent in the case of single-parent families). Categories were “native” for children with both parents born in Belgium, “2nd-generation migrant” for children born in Belgium and with at least one parent born abroad, and “1st-generation migrant” for children and parents born abroad. Both parents were asked about their employment status, which was categorized as “both working”, “one working parent”, and “parents not working”. A parent who was unemployed, sick, disabled or at home was considered as not working. Parents were also asked about their level of education, the highest in the household was retained and categorized as “secondary or lower”, “post-secondary short” and “post-secondary long”. For both work status and education, the answer “do not wish to answer” was considered as missing. Parents were asked to measure their child's weight and height, preferably at the time of completing the questionnaire, or to provide the most recent measurements, indicating whether they had been taken in the three previous months. BMI was calculated and categorized according to the age- and sex specific cut-offs recommended by the International Obesity Task Force (IOTF) [20].

For use in modelling usual intake (see below), we estimated whether children were likely to consume or never consume (i.e. “propensity”) water, SB, or milk, based on their response to the short beverage frequency questionnaire. SB consumption is a composite of four questions from this frequency questionnaire on “non-light soft drinks”, “light soft drinks”, “energy drinks” and “flavoured milk”, and another question on the permissibility of drinking fruit juices at home (i.e. children who answered “I do not like it/I never drink it” were considered as non-consumers of fruit juices).

### Statistical analyses and usual beverage consumption

All descriptive analyses were performed using Stata® version 17.0 Standard Edition (StataCorp, College Station, TX, USA). Logistic regressions were used to compare characteristics of schools and children included in the two different cohorts, *P* values < 0.05 considered statistically significant.

The “usual” consumption, i.e. the long-term average intake accounting for day-to-day variability, was estimated—along with 95% confidence intervals (CI) for total and three types of beverages by correcting the data for the within-person variation using the Statistical Program to Assess Dietary Exposure (SPADE version 4.1.35) [21, 22] and R (version 4.3.2). A 1-part model for daily consumed foods was used for total beverages, and a 2-part model for episodically consumed foods (i.e. > 10% of null daily consumptions in the dataset) for water (12.1% of null daily consumptions), SB (31.8%) and milk (74.4%). By default, the “non-consumers” (i.e. children known to never consume a beverage category based on their answers to beverage propensity questions) were included in the modelling, which was only possible for certain estimates of water and SB. The change of self-declared non-users into users was applied when they had a positive intake in the 4-day diary. Intakes were age-dependently modelled. In case of limited number of subjects in some subgroups, available information on non-consumers could not be taken into account, and age was considered as a constant. To correct for within-person variability in consumption, SPADE applies Box-Cox transformations and multi-stage Monte Carlo simulation models [21]. SPADE is preferably used with non-consecutive days to avoid underestimating intra-individual variability [22]. However, beverages tend to show higher day-to-day variation than food consumption and we ensured that the ratio of intra- to inter-individual variances was between 0.25 to 4.0 to support the reliability of the usual intake estimates. Such estimates could not be compared between student subgroups using usual statistical tests. We used the bootstrap method with 200 iterations, which generated 95% CIs. Differences between subgroups were considered as significant when their 95% CIs did not overlap.

## Results

### Participation of schools and reasons for non-participation

In total, 48 schools have been included in the trial baseline assessment (Fig. 1). School participation rates did not differ statistically according to the region (Wallonia: 32.7%; Brussels: 21.9%) or SEI (low SEI: 25.5%; moderate SEI: 32.8%; high SEI: 27.1%).

In the 92 non-participating schools with available information (among 120 non-participating schools), the main reasons for refusal were: availability and organizational issues (65.2%); past or current projects on beverage consumption (35.9%) or health promotion (7.6%); too many requests for this type of projects (7.6%); and lack of interest in the topic (6.5%). Additionally, more than eight out of ten declared that they had already taken measures to reduce the SB consumption such as encouraging students to bring water to school (66.3%), banning SB (54.4%), installing water fountains or renovating taps (15.2%), and promoting or mandating reusable bottles (9.8%).

### Cohort description at baseline

The staff from all the 48 included schools answered the school questionnaire, and 3,631 students from 46 schools were considered as having participated in the data collection, i.e. had validated either the 4-day diary, the child questionnaire, or the parent questionnaire.

#### Schools

In the 46 schools, the proportion of children included from schools with a low SEI was lower than the expected one-third (Table 1). The proportion of children from schools in the Brussels region fell from 27.9% with at least one valid questionnaire or a 4-day diary, to 11.5% with valid diary and questionnaires. Two-thirds of the data were collected in spring and summer, and one third in autumn and winter.

**Table 1:** Baseline characteristics of children from the 46 schools included in the DRINK^a^ trial, Belgium, 2021–22

	Total of participating students^b^ (*n* = 3 631)		4-day diary + child questionnaire completed (*n* = 2 427)		4-day diary + child questionnaire + parent questionnaire completed (*n* = 2 013)	
	n	%	n	%	n	%
School Region						
Brussels-Capital	1 013	27.9	397	16.4	232	11.5
Wallonia	2 618	72.1	2 030	83.6	1 781	88.5
School socio-economic index						
Low	601	16.6	433	17.9	350	17.4
Moderate	1 426	39.3	1,010	41.6	876	43.5
High	1 604	44.2	984	40.5	787	39.1
School size						
< 90 students	987	27.2	788	32.5	687	34.1
90–135 students	1 280	35.2	773	31.8	591	29.4
> 135 students	1 364	37.6	866	35.7	735	36.5
School network						
Public	1 973	54.3	1 293	53.3	1 067	53.0
Private	1 658	45.7	1 134	46.7	946	46.7
Year						
3rd	836	23.7	594	24.5	509	25.3
4th	1 309	37.2	885	36.4	721	35.8
5th^,c^	1 379	39.1	948	39.1	783	38.9
* missing*	*107*					
Season of 1st data collection						
Spring—summer	2 407	66.3	1 773	73.1	1 528	75.9
Autumn—winter	1 224	33.7	654	26.9	485	24.1

^a^Decreasing consumption of sugar-sweetened beverages and Raising tap water consumption through Interventions based on Nutrition and sustainability for Kids

^b^Students who at least have returned either the baseline valid 4-day diary, the child questionnaire or the parent questionnaire

^c^Including 13 students in 6th grade

**Fig. 2:**
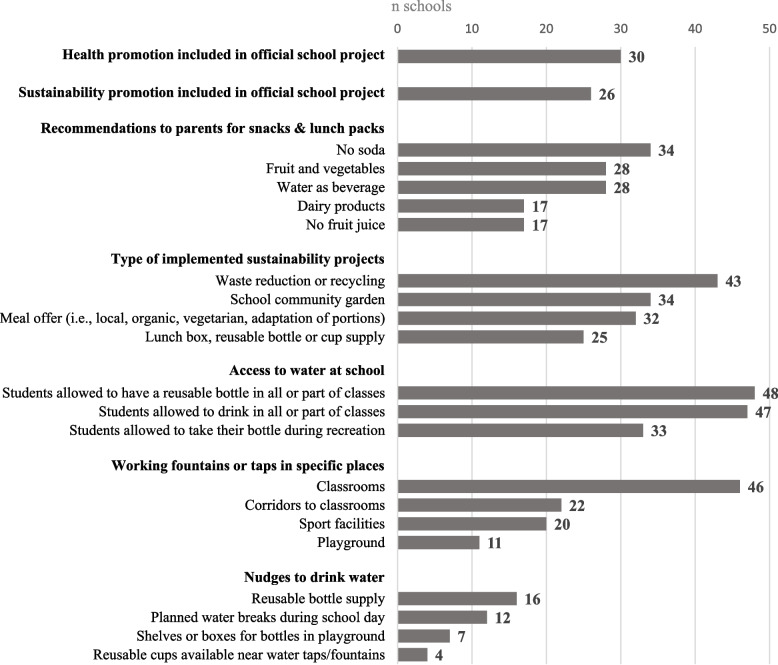
Health and sustainability promotion projects in the 48 schools included in the DRINK^*^ trial, Belgium, 2021–22. The schools responded to the baseline school staff interview prior to randomisation (n schools responding “yes”). ^*^Decreasing consumption of sugar-sweetened beverages and Raising tap water consumption through Interventions based on Nutrition and sustainability for Kids

Most of the 46 participating schools declared that they had included, prior to the DRINK trial, health and sustainability promotion in their official school project and developed nutritional and sustainable development actions (Fig. 2). Over three-quarters of schools advised parents against including sodas in children's meals, and one-third discouraged fruit juice; over half recommended water instead. Most schools had implemented recycling initiatives, and half had distributed reusable containers. While water access was common, facilities and incentives to encourage drinking water were less widespread (Fig. 2).

#### Students

Most of the included children were 9 to 10 years-old, living in families of a medium FAS, born in Belgium, had siblings and two working parents, regardless of the data completion sample considered (Table 2**)**. The proportion of girls was higher than boys. Among the 1,675 children with weight and height measured less than three months prior to data collection, 3.3% were thin, 80.1% had a normal weight, 13.4% were overweight, and 3.2% were obese.

**Table 2:** Baseline characteristics of children by completion of questionnaires and 4-day diary, DRINK^a^ trial, Belgium, 2021–22

	Total number of participating students^b^ (*n* = 3 631)		4-day diary + child questionnaire completed (*n* = 2 427)		4-day diary + child questionnaire + parent questionnaire completed (*n* = 2 013)	
	n	%	n	%	n	%
Age						
7–8 years	423	12.8	318	13.1	279	13.9
9 years	1 211	36.7	869	35.8	710	35.2
10 years	1 102	33.4	825	34.0	692	34.4
11–13 years	565	17.1	415	17.1	333	16.5
* missing*	*330*					
Sex						
Boy	1 490	47.7	1 072	44.2	872	43.3
Girl	1 632	52.3	1 352	55.8	1 141	56.7
* missing*	*509*		*3*			
Family Affluence Scale						
Low	433	14.4	301	12.9	231	11.8
Medium	1 894	63.0	1 507	64.4	1 268	65.1
High	680	22.6	531	22.7	450	23.1
* missing*	*624*		*88*		*64*	
Country of birth						
Belgium	2 649	88.2	2 104	89.2	1 794	91.3
Other country	355	11.8	255	10.8	170	8.7
* missing*	*627*		*68*		*49*	
Migration status						
Native	1 370	67.6	1 223	68.6	1 223	68.6
2nd-generation immigrant	540	26.7	466	26.1	466	26.1
1st-generation immigrant	116	5.7	94	5.3	94	5.3
* missing*	*1 605*		*645*		*231*	
Siblings						
Yes	2 724	90.5	2 132	90.2	1 770	90.0
No	286	9.5	231	9.8	197	10.0
* missing*	*621*		*64*		*46*	
Living with both parents						
Yes	2 170	72.1	1 732	73.3	1 452	73.9
No	840	27.9	630	26.7	514	26.1
* missing*	*621*		*65*		*47*	
Parental working status^c^						
Both working	1 597	66.3	1 254	68.1	1 254	68.1
One working parent	694	28.8	504	27.4	504	27.4
Parents not working	119	4.9	82	4.5	82	4.5
* missing*	*1 221*		*587*		*173*	
Parental highest education level^d^						
Secondary or lower	889	35.3	649	33.8	649	33.8
Post-secondary short	920	36.6	730	38.1	730	38.1
Post-secondary long	707	28.1	540	28.1	540	28.1
* missing*	*1 115*		*508*		*94*	

^a﻿^Decreasing consumption of sugar-sweetened beverages and Raising tap water consumption through Interventions based on Nutrition and sustainability for Kids

^b﻿^Students who at least have returned either the baseline valid 4-day diary, the child questionnaire or the parent questionnaire

^c﻿^*n* = 123 answers “do not wish to reply” from mothers and *n* = 118 from fathers were considered as missing

^d﻿^*n* = 116 answers “do not wish to reply” from mothers and *n* = 116 from fathers were considered as missing

### Usual beverage consumption and drink behaviours

Based on the data collected in the 4-day diary, the usual mean consumption of total beverages was 1,109 ml/day (95% CI: 1,092–1,132), including 677 ml/day (660–699) of water, 345 ml/day (331–359) of SB, and 60 ml/day (56–64) of non-sweetened milk (Tables 3 and 4). The beverage consumption also included 9 ml/day of coffee and tea, and 14 ml/day of soup, corresponding to 0.8% and 1.3% of the daily intake respectively (data not tabulated). Children’s usual beverage consumptions differed according to their demographic and socio-economic characteristics (Tables 3 and 4). A higher parental education level was associated with higher usual intake of total beverages, water and milk, and a lower usual SB intake. More favourable FAS was also associated with higher usual consumption of total beverages and water (Table 3). Moreover, children born in Belgium usually consumed greater amounts of total beverages and water than those born in another country (Table 3). Children whose parents both worked consumed higher amounts of water and lower amounts of SB than children with only one working parent (Tables 3 and 4). Children living with both parents usually consumed higher amount of water than those living in a one-parent or a blended family (Table 3). At last, higher amounts of unsweetened milk intakes were observed in the youngest children (Table 4).

**Table 3:** Children’s usual total beverage and water consumptions, baseline of the DRINK^a^ trial, Belgium, 2021–22

	Total beverages^b,c^ (ml/day)						Water^c,d^ (ml/day)					
	n	Mean	95% CI	P5	P50	P95	n	Mean	95% CI	P5	P50	P95
Usual daily consumption	2 233	1 109	1 092–1 132	526	1 067	1 836	2 165	677^e^	660–699	203	627	1 320
Age	2 233						2 165					
7–8 years		1 106	1 066–1 164	526	1 061	1 830		686^e^	647–737	210	638	1 331
9 years		1 111	1 084–1 135	527	1 070	1 837		676^e^	652–703	204	625	1 318
10 years		1 110	1 086–1 135	527	1 068	1 840		676^e^	656–698	201	627	1 319
11–13 years		1 107	1 075–1 159	524	1 061	1 832		674^e^	639–716	201	624	1 318
Sex	2 233						2 165					
Boy		1 112	1 086–1 144	541	1 071	1 822		687^e^	654–717	203	644	1 316
Girl		1 107	1 083–1 133	515	1 063	1 848		672^e^	650–698	205	620	1 321
Family Affluence Scale	2 149						2 091					
Low		1,032^f^	965–1 083^f^	438	977	1 809		584^f^	535–636^f^	152	542	1 157
Medium		1,114^f^	1 090–1 142^f^	539	1 075	1 825		670^f^	647–693^f^	200	620	1 310
High		1,158^f^	1 115–1 200^f^	563	1 118	1 896		745^f^	699–787^f^	241	696	1 412
Country of birth	2 169						2 106					
Belgium		1 124^f^	1 103–1 148^f^	538	1 084	1 845		691^f^	671–713^f^	208	641	1 341
Other country		1 029^f^	964–1 091^f^	484	984	1 732		611^f^	560–664^f^	194	568	1 176
Siblings	2 173						2 110					
Yes		1 108	1 087–1 128	526	1 066	1 832		681	658–702	202	630	1 330
No		1 174	1 117–1 234	599	1 146	1 844		704	645–758	234	664	1 314
Living with both parents	2 172						2 109					
Yes		1 123	1 100–1 148	542	1 083	1 840		698^f^	676–721^f^	215	649	1 344
No		1 086	1 042–1 128	493	1 040	1 827		634^f^	594–670^f^	177	583	1 260
Parental working status	1 701						1 656					
Both working		1 146	1 117–1 174	546	1 105	1 879		727^f^	703–754^f^	227	678	1 394
One working parent		1 099	1 058–1 142	548	1 061	1 781		615^f^	575–651^f^	199	572	1 170
Parents not working		1 137	1 018–1 254	424	1 071	2 094		636	548–740	97	578	1 369
Parental highest education level	1 770						1 719					
Secondary or lower		1 072^f^	1 034–1 111^f^	519	1 030	1 764		591^f^	556–622^f^	186	557	1 109
Post-secondary short		1 156^f^	1 122–1 188^f^	565	1 118	1 876		712^f^	678–746^f^	198	659	1 401
Post-secondary long		1 154^f^	1 119–1 199^f^	556	1 115	1 888		774^f^	736–818^f^	280	726	1 432

*CI* Confidence interval, *P5-P50-P95* Percentiles, *SD* Standard deviation

^a^Decreasing consumption of sugar-sweetened beverages and Raising tap water consumption through Interventions based on Nutrition and sustainability for Kids

^b^Children who completed the 4-day booklet, and for which age and sex and subgroup variables were not missing

^c^Excluding over-reporters: total water > mean + 2 SD (i.e. > 2 060 g/day) and total beverages > mean + 2 SD (i.e. > 2 366 g/day) = 8%

^d^Children who completed the 4-day booklet, for which age and sex and subgroup variables were not missing, and who responded to the beverage propensity questionnaire

^e^To distinguish “true” non-consumers from occasional consumers, the estimated usual intake took into account the propensity to consume or never consume water (0.7% of non-consumers)

^f^Non-overlapping 95% CIs, indicating a significant difference between subgroups

**Table 4:** Children’s usual sweetened beverage and non-sweetened milk consumptions, baseline of the DRINK^a^ trial, Belgium, 2021–22

	Sweetened beverages^b,c^ (ml/day)						Non-sweetened milk^c,d^ (ml/day)					
	n	Mean	95% CI	P5	P50	P95	n	Mean	95% CI	P5	P50	P95
Usual daily consumption	2 099	345^e^	331–359	57	311	749	2 233	60	56–64	0	24	223
Age	2 099						2 233					
7–8 years		322^e^	296–363	51	287	710		70^f^	59–82^f^	0	35	243
9 years		338^e^	314–354	54	304	734		63^f^	57–70^f^	0	27	227
10 years		359^e^	335–375	66	324	769		59	54–65	0	24	221
11–13 years		352^e^	325–393	53	317	768		48^f^	40–55^f^	0	12	200
Sex	2 099						2 233					
Boy		333^e^	312–354	40	296	752		66	59–71	0	28	236
Girl		355^e^	336–372	71	322	749		56	51–61	0	22	214
Family Affluence Scale	2 028						2 149					
Low		360	320–396	81	322	765		58	44–70	0	27	215
Medium		353	336–370	62	318	762		62	57–67	0	25	229
High		333	303–365	48	297	744		57	48–66	0	22	216
Country of birth	2 043						2 169					
Belgium		345	330–359	58	311	749		60	57–64	0	23	226
Other country		334	295–379	75	297	802		58	48–70	0	31	200
Siblings	2 044						2 173					
Yes		340	326–354	60	305	739		60	56–65	0	25	225
No		385	340–430	85	352	802		59	46–71	0	20	223
Living with both parents	2 048						2 172					
Yes		336^e^	321–353	49	304	734		62	58–66	0	25	228
No		371^e^	342–398	76	333	795		54	46–60	0	21	206
Parental working status	1 614						1 701					
Both working		329^f^	311–344^f^	51	296	718		64	59–71	0	25	240
One working parent		393^f^	368–429^f^	82	355	832		58	49–68	0	24	217
Parents not working		414	331–486	113	382	832		62	36–71	0	34	214
Parental highest education level	1 672						1 770					
Secondary or lower		404^f^	374–434^f^	71	361	889		54^f^	47–60^f^	0	21	204
Post-secondary short		355^f^	330–379^f^	64	322	755		61	53–69	0	24	228
Post-secondary long		279^f^	253–300^f^	46	256	590		73^f^	63–81^f^	0	34	255

*CI* Confidence interval, *P5-P50-P95* Percentiles, *SD* Standard deviation

^a^Decreasing consumption of sugar-sweetened beverages and Raising tap water consumption through Interventions based on Nutrition and sustainability for Kids

^b^Children who completed the 4-day booklet, for which age and sex and subgroup variables were not missing, and who responded to the beverage propensity questionnaire

^c^Excluding over-reporters: total water > mean + 2 SD (i.e. > 2 060 g/day) and total beverages > mean + 2 SD (i.e. > 2 366 g/day) = 8%

^d^Children who completed the 4-day booklet, and for which age and sex and subgroup variables were not missing

^e^To distinguish “true” non-consumers from occasional consumers, the estimated usual intake took into account the propensity to consume or never consume sweetened beverages (including soft drinks, energy drinks, flavoured milk, and fruit juices; 0.8% of non-consumers)

^f^Non-overlapping 95% CIs, indicating a significant difference between subgroups

## Discussion

The present analyses highlighted a broad variety in the characteristics of the schools and children who participated in the baseline collection of the DRINK trial. In addition, socio-economic disparities in the children’s usual beverage consumption were observed, mostly related to the parental education, and most strikingly for the water intake.

### Characteristics of schools and children included

A large variety of schools and of children were eventually included in the trial consistently with the protocol goals [13]. Most of the schools were already active in the fields of nutritional health promotion and environmental sustainability but with variability in the nature, extent and continuity in actions implemented. Such a diversity of contexts, often unrepresented or undocumented in previous interventional studies [10, 23], is an asset for evaluating and scaling up interventions. However, the participation of the invited schools was lower than expected, and although these differences were not significant, it was lower in Brussels and in the more and the less socio-economically advantaged schools. In addition to the recruitment difficulties encountered in the school context [14], the stringent COVID-19 consequences were still ongoing when the schools were invited to participate in the DRINK trial. Additionally, children from disadvantaged socio-economic backgrounds may have had difficulty reading and responding to questionnaires and receiving sufficient adult support to participate assiduously in the beverage collection [24]. These circumstances help understand the differences in the characteristics of children according to the questionnaire and 4-day diary completion.

While, in 2020–21, 49.4% of the French-speaking primary school population were girls [25], a higher proportion of girls than boys participated in the baseline DRINK data collection, this difference being wider among children with all valid questionnaires. Girls might have been more eager to complete the questionnaire and diary, consistently with their school task engagement while boys are considered to behave in a more disruptive way [26]. Furthermore, the proportion of disadvantaged children was lower among those who completed the three instruments compared to all participating children [27]. Such under-representation will be considered in the further analyses of the overall trial, by adjusting on the SEI and gender.

Nonetheless, the proportions of children in the different categories of body weight status were consistent with previous observations, similarly based on the IOTF thresholds [20], and despite the use of height and weight reported by parents in the DRINK trial. Recent national surveys based on self-reported [28] or measured [29] weight and height show consistent patterns with our findings, supporting the relevance and validity of the weight status in our sample. An underestimation of body weight status is observed when it is reported by parents, either by the parents or the students [30, 31], but findings in the DRINK trial showed non-additional bias.

Thus, despite some challenges, especially in relation with the period when it was initiated, the inclusion in the DRINK trial led to a broad diversity of school population. However, the partial questionnaire and diary completion, will raise methodological issues for the main trial analyses.

### Usual consumption of beverages

Another challenge in the DRINK trial as others addressing similar topics [23, 32], is to rely on trustworthy beverage consumption estimates. The European Food Safety Authority (EFSA) [33] and the Belgian recommendations [34] for water intake primarily target adults and, therefore, are not directly applicable for comparing our estimations. Moreover, the recommendation for adults regarding sugar-sweetened beverages [35] is simply to “limit” their consumption, without specifying a threshold, which hinders a nuanced comparison with our data. Nevertheless, our findings were in line with those reported in the 2014 BNFCS, which is based on the EFSA recommended protocol [36], regarding the usual consumptions of “non-alcoholic beverages” and water by boys and girls aged 10–13 years [18]. Using data collection and analysis methods comparable to the BNFCS, the total beverage intake in the Netherlands in 2012–2016 [37] and water intakes in France in 2014–2015 [38], i.e. in neighbouring countries of Belgium, were also similar to the estimates presented here. Overall, the reliability of the 4-day diary for assessing usual total and water beverage consumption in children is supported and the future generalizability of the trial findings is reinforced.

The categorization of SB used in the present study was aligned with the trial’s objectives, which focused on sweet-tasting beverages from nutritional but also behavioural points of view. The definition of the SB group in the other surveys differed from this approach, hindering the direct comparisons to the corresponding estimates. However, the usual consumption of SB in the present study was also in the range observed in the European national food consumptions surveys [18, 39]. Yet again, these observations support the plausibility of the consumptions estimated in the DRINK trial, despite the selection bias in schools and participants.

### Beverage disparities

At baseline in the DRINK trial, usual beverage consumption was more favourable to health, i.e. higher total, water and milk, and lower SB intake, when the parental school achievement was higher. Indeed, the overall diet quality of adolescents has been previously shown to be associated to parental education, more consistently than to parental occupation or income [5]. Children and adolescents of lower parental education are more likely to drink lower amounts of water or to not consume water daily [16, 40, 41], and larger quantities of SB or to consume them daily [5, 16, 40, 41]. Higher dairy (including milk) intake has also been associated with a higher parental education level, but less consistently across studies [5]. Our findings confirm that parental educational background remains a strong determinant of healthier beverage consumption in children. Possible mechanisms include raised cognitive and emotional skills, and the development of health literacy in relation with the educated social network [42]. Such findings support the need to integrate parental information and literacy levels when developing nutrition interventions for young students, especially for those living in poorly educated families [43, 44].

In the DRINK baseline assessment, material affluence, family structure, and country of birth were also associated to variations in the usual beverage consumption. While they are acknowledged to be interrelated [5, 42], these factors may have a cumulative effect, and such complex pathways remain to be fully understood. This would require exploring consumptions in multivariable models, a different purpose of the analyses here, which were based on the usual consumption modelling [21].

Possible mechanisms and mediators of diet disparities in children, previously described in various contexts [44, 45], involve individual and familial characteristics along with schooling, larger environment of living, and policy dimensions [43, 44, 46, 47]. For instance, parental education differences in soft drink intake by Norwegian 12-year-old adolescents were explained by various mediators such as parental modelling, SB accessibility at home (especially when living in low-income neighbourhood), and purchase frequency [48]. Total beverage and water disparities may also be determined by parental health literacy and socio-economic environment, like household wealth, living conditions, structure and country of origin [5, 42, 49]. Beyond the family nucleus, and additionally to their high taste attractiveness for the young children [50], the intensive marketing targeted at children and adolescents and their high accessibility and affordability have been shown to determine children’s SB consumption [4, 10]. Other relevant determinants could have been investigated, and some were available in our dataset; however, a broader analysis was beyond the scope of this article.

Socio-economic disparities in health, exacerbated during the pandemic [51], may have impacted dietary behaviours [52], including beverage consumption. They must be considered when developing actions in the school setting, as well as in the frame of such a trial analysis.

### Strengths and limitations

By using a stratified random sample of schools, the different strata of region and SEI were well represented overall, despite a lower participation rate than expected but comparable to other cross-sectional studies in a similar context [53]. Although the study was not designed for surveillance purposes but rather to develop a cohort with planned interventions, the consumption estimates obtained remain highly consistent with those from national surveillance surveys [18, 37, 38], which supports the reliability of our findings. Nevertheless, to obtain a better participation in the data completion by most children, on-site support would have been necessary. In fact, some of the questions could have been difficult for their age, and despite the completion in class, the teachers, were not necessarily willing to help them. The parents’ questionnaire, distributed through their children, contributed to obtain the most difficult information. However, the loss of returned questionnaires and refusals may have introduced a selection bias. The interviews with school staff, although participation was complete, may have been subject to response bias due to the respondents’ awareness of the study topic and the fact that the interviewers were dieticians. Future analyses will allow an in-depth understanding of the variability of school situations.

Modelling the usual consumption (SPADE) [21] should normally be used for amounts of beverages consumed on several but independent days, such as repeated 24-h recalls [21]. Nevertheless, the 4-day diary enabled to include days that may differ in terms of drinking behaviour, such as Wednesdays (half day off school) or weekend days, which has enhanced the estimates of individual variations. The predefined amounts of beverage consumed on the stickers, despite their wide range, may have led to over-reporting. Such misreporting has been rigorously identified to be corrected or excluded from analyses. In contrast, difficulties in memorization, lack of motivation to maintain participation throughout the four days of collection, and social desirability related to beverage categories such as SB, may have led to under-reporting, globally or for some specific beverages. Nevertheless, the mean SB amounts estimated here remained very high. Finally, despite corrections and due to the episodic nature of consumption, the distributions of water, SB, and milk consumption obtained were wide. We therefore used usual consumption modelling to correct such remaining within-person variations and were able to robustly compare mean usual consumptions between socio-economic subgroups. It is note worthy that this modelling approach imposed to consider differences significant only when 95% confidence intervals do not overlap, and while conservative, this may underestimate certain disparities, e.g., for milk consumption by sex or family structure, where intervals overlapped by only 2 ml.

Although its careful design and pre-testing, the 4-day diary lacks formal validation in children and may be affected by recall or social desirability bias. The calibration study, conducted in a sub-sample with dieticians’ interviews, will help refine analyses of the trial findings.

## Conclusion

The primary schools and students who participated in the first data collection of the DRINK trial were diverse. However, the socioeconomic disparities observed in the completion of the questionnaire should ultimately be taken into account when interpreting the subsequent trial findings. The robust methodology of data collection and analysis provides estimates of children’s usual beverage consumption that can be considered as reliable given their consistency with methods of reference. In addition, beverage consumption varied according to the socio-economic characteristics, underlining the importance of involving nutrition professionals in schools and the relevance of developing targeted strategies for disadvantaged groups based on the determinants identified here.

## Data Availability

The datasets analysed during the current study are available from the corresponding author on reasonable request.

## Abbreviations

BNFCS: Belgian food consumption survey
DRINK: Decreasing consumption of sugar-sweetened beverages and Raising tap water consumption through Interventions based on Nutrition and sustainability for Kids
EFSA: European Food Safety Authority
FAS: Family affluence scale
IOTF: International obesity task force
SB: Sweetened beverages
SEI: Socio-economic index
SPADE: Statistical program to assess dietary exposure
